# Potential DNA barcodes for *Melilotus* species based on five single loci and their combinations

**DOI:** 10.1371/journal.pone.0182693

**Published:** 2017-09-14

**Authors:** Fan Wu, Jinxing Ma, Yuqin Meng, Daiyu Zhang, Blaise Pascal Muvunyi, Kai Luo, Hongyan Di, Wenli Guo, Yanrong Wang, Baochang Feng, Jiyu Zhang

**Affiliations:** 1 State Key Laboratory of Grassland Agro-ecosystems, College of Pastoral Agriculture Science and Technology, Lanzhou University, Lanzhou, China; 2 National Quality Control & Inspection Centre for Grassland Industry Products, National Animal Husbandry Service, Ministry of Agriculture, Beijing, China; 3 China Agricultural Veterinarian Biology Science and Technology Co. Ltd, Lanzhou, China; Chinese Academy of Medical Sciences and Peking Union Medical College, CHINA

## Abstract

*Melilotus*, an annual or biennial herb, belongs to the tribe Trifolieae (Leguminosae) and consists of 19 species. As an important green manure crop, diverse *Melilotus* species have different values as feed and medicine. To identify different *Melilotus* species, we examined the efficiency of five candidate regions as barcodes, including the internal transcribed spacer (ITS) and two chloroplast loci, *rbc*L and *mat*K, and two non-coding loci, *trn*H-*psb*A and *trn*L-F. In total, 198 individuals from 98 accessions representing 18 *Melilotus* species were sequenced for these five potential barcodes. Based on inter-specific divergence, we analysed sequences and confirmed that each candidate barcode was able to identify some of the 18 species. The resolution of a single barcode and its combinations ranged from 33.33% to 88.89%. Analysis of pairwise distances showed that *mat*K+*rbc*L+*trn*L-F+*trn*H-*psb*A+ITS (MRTPI) had the greatest value and *rbc*L the least. Barcode gap values and similarity value analyses confirmed these trends. The results indicated that an ITS region, successfully identifying 13 of 18 species, was the most appropriate single barcode and that the combination of all five potential barcodes identified 16 of the 18 species. We conclude that MRTPI is the most effective tool for *Melilotus* species identification. Taking full advantage of the barcode system, a clear taxonomic relationship can be applied to identify *Melilotus* species and enhance their practical production.

## Introduction

*Melilotus* (sweet clover), an annual or biennial herb, belongs to the tribe Trifolieae (Leguminosae) and consists of 19 species mainly distributed in North Africa and Eurasia [1]. These species can endure extreme environmental conditions, such as high salinity, drought and cold [1, 2], and have important medicinal value [3]. Coumarin is an important plant secondary metabolism compound found in *Melilotus* [4] and possesses a several antitumor activities, both preventing the occurrence of cancer and potentially curing cancer [5]. However, it is difficult to assess the value of different species, as the coumarin content varies, with the highest (0.943%) in *M*. *indicus* accessions and none in *M*. *segetalis* accessions [6]. Among *Melilotus* species, characters of leaf, flower colour and structure, pod and seed present extensive variations [7], and although traditional classification methods can distinguish different *Melilotus* species, only experts and those with experience can accurately identify them. However, DNA barcodes can be used to rapidly identify different plants without extensive expertise. DNA barcode analysis examining one or several brief and standardized DNA fragment(s) [8, 9], allows for rapid, exact taxon discrimination. The primers utilized for DNA barcodes should be applied to the widest taxonomy, and standardized sequences 500–800 bp in length are used to distinguish among species from all eukaryotic kingdoms [10]. Ideally, a barcode region is stable, is particular to one species, and exhibits ample variation at the locus among species but little variation within a species. Thus, one can use such a sequence to unequivocally to identify closely related species [9, 11]. Although the universal barcode cytochrome c oxidase subunit 1 (*CO1*) is suitable for animals [9], it cannot be used as a barcode for plants given the extremely low mutation rate and unstable structure of the *CO1* region in plant genomes [12].

So far, many studies have been taken in search of the universal plant barcode and several loci have been suggested as DNA barcodes in plants. These studies usually comprised wide taxonomic units, including Dicotyledonous plants, family of Fabaceae, tribe Trifolieae and so on. ITS2 region was used to identified dicotyledons, monocotyledons, gymnosperms and ferns, the success rates was different [13]. Fabaceae is a huge family, among them 91.3% of 104 Fabaceae medicinal species was identified successfully by using of *trn*H*-psb*A sequences [14]. Data from *trn*H*-psb*A region were analysed to illuminate molecular evolution of Maghrebian *Medicago* species and reveal high interspecific diversity and low intraspecific variation [15]. Three cpDNA regions (*rbc*L, *trn*H*-psb*A and *mat*K) can distinguish Vachellia genera and discriminate sister-species among populations from Africa, Australia and India [16]. Besides, DNA barcodes were used to identify set of taxa characteristic for a certain region and analyzed as “local flora”. Costion and colleagues employed three types of barcodes (*rbc*La, *mat*K and *trn*H-*psb*A) to produce a DNA barcode reference library for Australian tropical plants [17]. Inter-phylogenetic information and evolutionary history of trees in Puerto Rico were obtained using three DNA barcodes (*rbc*L, *mat*K and *trn*H*-psb*A) [18]. DNA barcodes were also applied to other different species. For the identification of medicinal plants, the internal transcribed spacer 2 (ITS2) sequence combined with the *psb*A-*trn*H sequence was recommended as one of most suitable DNA barcode [19, 20], and ITS2 was used to accurately distinguish medicinal plants in *Artemisia* [21]. Nuclear ITS sequence data can also be utilized to provide new information for identifying poisonous mushroom species [22] and to study the genetic diversity of *M*. *albus* and *M*. *officinalis* [23].

Previous researches have revealed that core barcodes, some combinations of potential barcodes, standard markers, and other sequences are not sufficiently reliable for DNA barcode development [24, 25]. Despite the broad applications of these markers, mutation rates of single-locus barcodes can be low, and certain regions, such as *trn*H-*psb*A, demonstrate amplification problems. Thus far, some multigene methods have been proposed to apply combinations of plastid regions that are relatively conservative as well as coding and non-coding fragments [26, 27]. In addition, the majority of studies at the genus level have only involved a small proportion of species [28, 29] and either concentrated on a single species [30] or focused on discovering a single universal barcode [10]. Therefore, standard barcodes for discriminating plant species are associated with several challenges and it is very difficult to reconcile with barcode universality.

For the study of barcode, there are only few closely related species representing the same genus, rather than focus on species identification in the case of very closely related taxa. In our study, we concerned barcode analysis of *Melilotus* at the level of species. Regarding the 18 *Melilotus* species examined in this study, we are quite confident about the materials because we analysed 5 seed morphological traits and 9 agronomic traits for these germplasms [31, 32]. Moreover, 40 half-sib (HS) families of *M*. *officinalis* were obtained to evaluate genotypic variation as well as phenotypic and genotypic correlations [33]. Simple sequence repeat (SSR) analysis was performed to evaluate the genetic diversity of the 18 *Melilotus* species [34], and phylogenetic trees were constructed to study their inter-specific relationships [32]. No studies to date have measured the resolution of the DNA barcode system using numerous specimens covering almost all *Melilotus* species, and we established a standard DNA barcode system to assess the discrimination ability of each to propose the most powerful potential barcodes for *Melilotus*. The loci were selected based on the following two major criteria: a high level of species identification with broad coverage and a high-quality sequence. We used 201 individuals representing 18 *Melilotus* species to compare barcode performance for a nuclear locus (ITS), four plastid markers (*trn*H-*psb*A, *trn*L-F, *mat*K, *rbc*L) and five combinations based on analysis of the barcode gap, similarity and pairwise distance.

## Materials and methods

### Plant materials

Seeds were selected from 98 accessions (S1 Table) representing 18 *Melilotus* species from Nation Plant Germplasm System (NPGS, USA) [32] and National Gene Bank of Forage Germplasm (NGBFG, China). Two to three accessions were selected to represent each of the *Melilotus* species. Prior to cultivation, seeds were gently polished and incubated at 24°C for 16 and 8 hours of light and darkness, respectively. After 10 days of cultivation, 20 fresh seedlings from each accession were collected seperatly, frozen in liquid nitrogen and stored at -80°C until assayed.

### DNA extraction, amplification, and sequencing

For each sample, total genomic DNA was extracted from whole seedlings using the SDS (sodium dodecyl sulfate) method [35]. Five pairs of primers, the internal transcribed spacer (ITS) [32], two chloroplast loci, *rbc*L [36] and *mat*K [37], and two non-coding regions, *trn*H-*psb*A [38] and *trn*L-F [39], were amplified and sequenced. A standard polymerase chain reaction (PCR) in a volume of 25 μL was prepared as follows: 12.25 μL 2×Reaction Mix, 0.25 μL Golden DNA Polymerase, 2 μL each primer (10 μmol/mL), 6.5 μL ddH_2_O and 2 μL template genomic DNA (50 ng/mL). The PCR program was as follow: 94°C for 3 min for pre-denaturation; 35 cycles of denaturation for 30 s at 94°C, annealing for 30 s at 53°C, and extension for 50 s at 72°C, with the annealing temperature and extension time varying according to the different barcode genes, see Table 1; and a final extension for 7 min at 72°C and a hold at 4°C. Amplicons were sequenced by Shenggong Biotechnological, Ltd (Shanghai, China). Successfully sequenced samples were recorded.

**Table 1:** Information for PCR primers and amplification conditions used for five potential barcodes.

Regin	Primer sequence(5′-3′)	Tm(°C)	Extension(s)	Aligned sequence length(bp)	The head of sequence	The end of sequence
ITS	F:GGAAGKARAAGTCGTAACAAGG	53	50	646	CCAACACGTGAATCAGTTTGAACAC	GGGGCTACCCGCTGAATTTAAGCAT
	R:RGTTTCTTTTCCTCCGCTTA					
*psb*A-*trn*H	F:GTTATGCATGAACGTAATGCTC	60	40	317	CTGCGGTCGAGGCTCCATCTATAAA	TGGATTTGTGAATCCCCATGCGCGA
	R:CGCGCATGGTGGATTCACAAATC					
*mat*K	F:CCCRTYCATCTGGAAATCTTGGTTC	52	60	713	TTCATTTATTACGATTGTTTCTTTA	CGATTTTTGCGAATATGCAGAATCT
	R:GCTRTRATAATGAGAAAGATTCTGC					
*trn*L-F	F:CGAAATCGGTAGACGCTACG	53	55	431/642	CTTACCAAGTGAAAACTTTCAAATT	CGGTAAAGCAAAGGACTGAAAATCC
	R:ATTTGAACTGGTGACACGAG					
*rbc*L	F:AGACCTWTTTGAAGAAGGTTCWGT	53	50	754	CGCGCTCTACGTCTGGAAGATTTGC	GTTCTGCCTGTTGCTTCGGGTGGTA
	R:TCGGTYAGAGCRGGCATRTGCCA					

ITS, internal transcribed spacer

### Alignment

Contigs were assembled and edited prior to alignment. The Contig Express module of Vector NTI Suite 6.0 software was used to remove both ends of the sequences and to keep the head and tail of the same gene at homologous sites. Sequences were aligned using DNAMAN6.0.

### Single-barcode analyses

Sequence alignment for the five DNA regions was performed using ClustalW of MEGA 6.0 software [40]. The Neighbour-Joining (NJ) method was used to generate a phylogenetic tree to obtain a pre-estimate of the discrimination ability of the five barcodes. The number of differences was used, and bootstrap values were calculated for 1000 replicates during construction of the NJ tree. Inter-specific genetic pairwise distances were calculated by Computing Pairwise Distance using MEGA 6.0 software. The candidate barcodes were classified on the basis of their identification ability. The sequences for each potential barcode were aligned among pairs, and the Emboss Needle algorithm (http://www.ebi.ac.uk/Tools/psa/emboss_needle/nucleotide.html) was used to calculate the barcode gap value, score and similarity of each sequence. For comparison of gaps between accessions, 36 datasets were divided into two subsets according to species affinity, resulting in 18 sequences representing 18 species in each group.

### Combination-barcode analysis

Based on single candidate barcodes that were able to identify a few of 18 species, we assembled combinations of potential barcodes and obtained 195 combination sequences. These combinations included *mat*K+*rbc*L (MR), *mat*K+*rbc*L+*trn*H-*psb*A (MRP), *mat*K+*rbc*L+*trn*H-*psb*A+ITS (MRPI), *mat*K+*rbc*L+*trn*L+*trn*H-*psb*A (MRTP), and *mat*K+*rbc*L+*trn*L+*trn*H-*psb*A+ITS (MRTPI). The combinations of each accession were assembled such that all sequences were connected end to end in the same order (Table 2). The same methods described above were used to assess the combination barcodes.

**Table 2:** Results of comparison used to assess the utility of five potential barcodes.

	Universal primer	PCR success (%)	Sequencing success (%)	No. of SNPs	No. of indels	No. of species successfully identify
ITS	Yes	99	100	78	8	13
*psbA-trnH*	Yes	86	95	27	5	9
*matK*	Yes	100	99	26	0	9
*trnL-F*	Yes	100	87	25	3	12
*rbcL*	Yes	100	100	14	0	6

ITS, internal transcribed spacer

## Results

### Amplification, sequencing and alignment

We primarily searched for primers that can successfully amplify the five chosen DNA regions of 18 *Melilotus* species. We utilized five universal primers (Table 1), amplifying a 646-bp sequence of ITS, a 317-bp sequence of *trn*H*-psb*A, a 713-bp sequence of *mat*K, 431-bp and 462-bp sequences of *trn*L-F and a 754-bp sequence of *rbc*L. Except for *trn*H*-psb*A (86%), PCR amplification succeeded at rates of 99% to 100% among four of the potential barcodes. Moreover, the sequencing success rates ranged from 87% (*trn*L-F) to 100% (*mat*K) (Table 2).

A variation of 30 bp in length was noted in each barcode gene sequence evaluated. The highest number of single-nucleotide polymorphisms (SNPs) and indels was 78 and 8, respectively, for ITS; the lowest number of SNPs and indels was 14 and 0, respectively, for *rbc*L (Table 2).

After editing and aligning the sequences, 18 pairs of standardized barcode sequences for each barcode system were obtained (S1 Dataset).

### The gap and distance of accessions and individuals

In total, 881 sequences (non-combinations) from 18 *Melilotus* species were obtained. Among those sequences, 100 individuals and 70 accessions were sequenced, with some success. For *M*. *officinalis*, the average gap means of 20 individuals for ITS, *trnH-psbA*, *matK*, *trnl-F* and *rbcL* were 0.000%, 0.029%, 0.148%, 0.000% and 0.000%, respectively. The average gaps for *M*. *albus*, *M*. *latissimus*, *M*. *dentatus* and *M*. *spicatus* ranged from 0.000% (ITS, *trn*H*-psb*A, *mat*K, *rbc*L) to 1.083% (*trn*L-F) (S2 Table). The mean sequence distances of 20 individuals of *M*. *officinalis* were 0.63 (ITS), 0.25 (*trnH-psbA*), 1.28 (*matK*), 0.5 (*trnl-F*) and 0.00 (*rbcL*) (S2 Table). For accession sequences, both the gap and distance values were small: most of the gap and distance values were 0; the largest mean gap was 1.728% (*M*. *wolgicus*, *trnH-psbA*), and the largest mean distance was 4.02 (*M*. *wolgicus*, *trnH-psbA*) (S3 Table).

**Fig 1:**
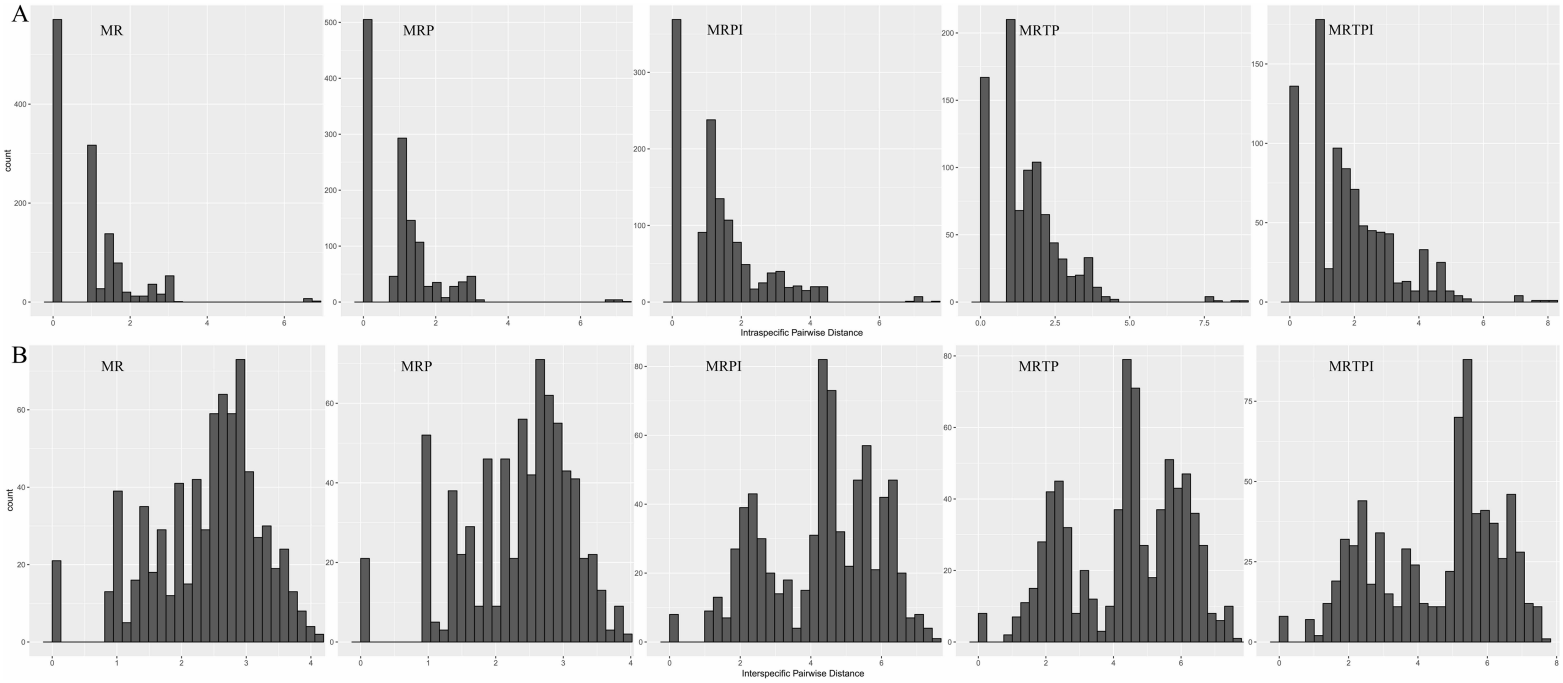
Barcode gap analyses using distance histograms for five combination barcodes. The dataset of the sequences was obtained from MEGA 6.0 Compute Pairwise Distance. The histograms plots generated using R 3.2.3 display intraspecific and interspecific variation. A. Intraspecific pairwise distance, B. interspecific pairwise distance. MR, *mat*K+*rbc*L; MRP, *mat*K+*rbc*L+*trn*H-*psb*A; MRPI, *mat*K+*rbc*L+*trn*H-*psb*A+ITS; MRTP, *mat*K+*rbc*L+*trn*L-F+*trn*H-*psb*A; MRTPI, *mat*K+*rbc*L+*trn*L-F+*trn*H-*psb*A+ITS.

After combining the five barcodes, intra-specific distances were found to be quite low, similar to the single-barcode values, with the majority of values being less than 1 (Fig 1A). The intra-species and intra-accession gap and distance values were similar to each other, with no apparent discrepancy.

Because each value for the five barcodes was based on different subsamples and randomly selected samples representing nine species, we decreased the number of sequences to 200, which fully covered the five gene regions and the 18 *Melilotus* species to enable species resolution comparisons among the single barcodes and their combinations.

### Single-barcode analysis

**Fig 2:**
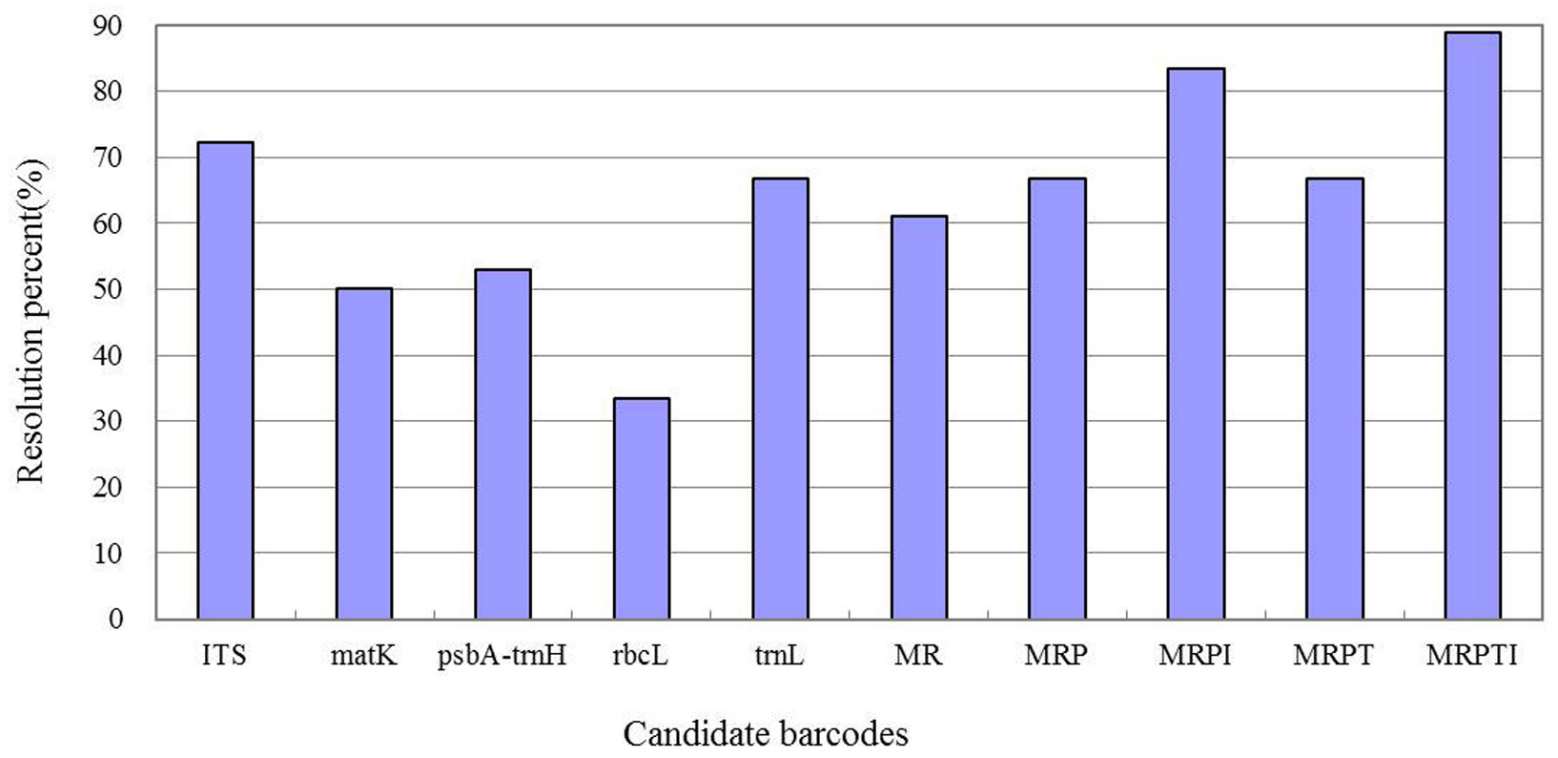
Percentages of species resolution for five single loci and their combinations. Specimens were analysed based on Neighbour-Joining in MEGA 6.0. The plots show the combinations of barcode loci surveyed on the x axis. I, ITS; M, *mat*K; R, *rbc*L; P, *trn*H-*psb*A; T, *trn*L-F.

**Fig 3:**
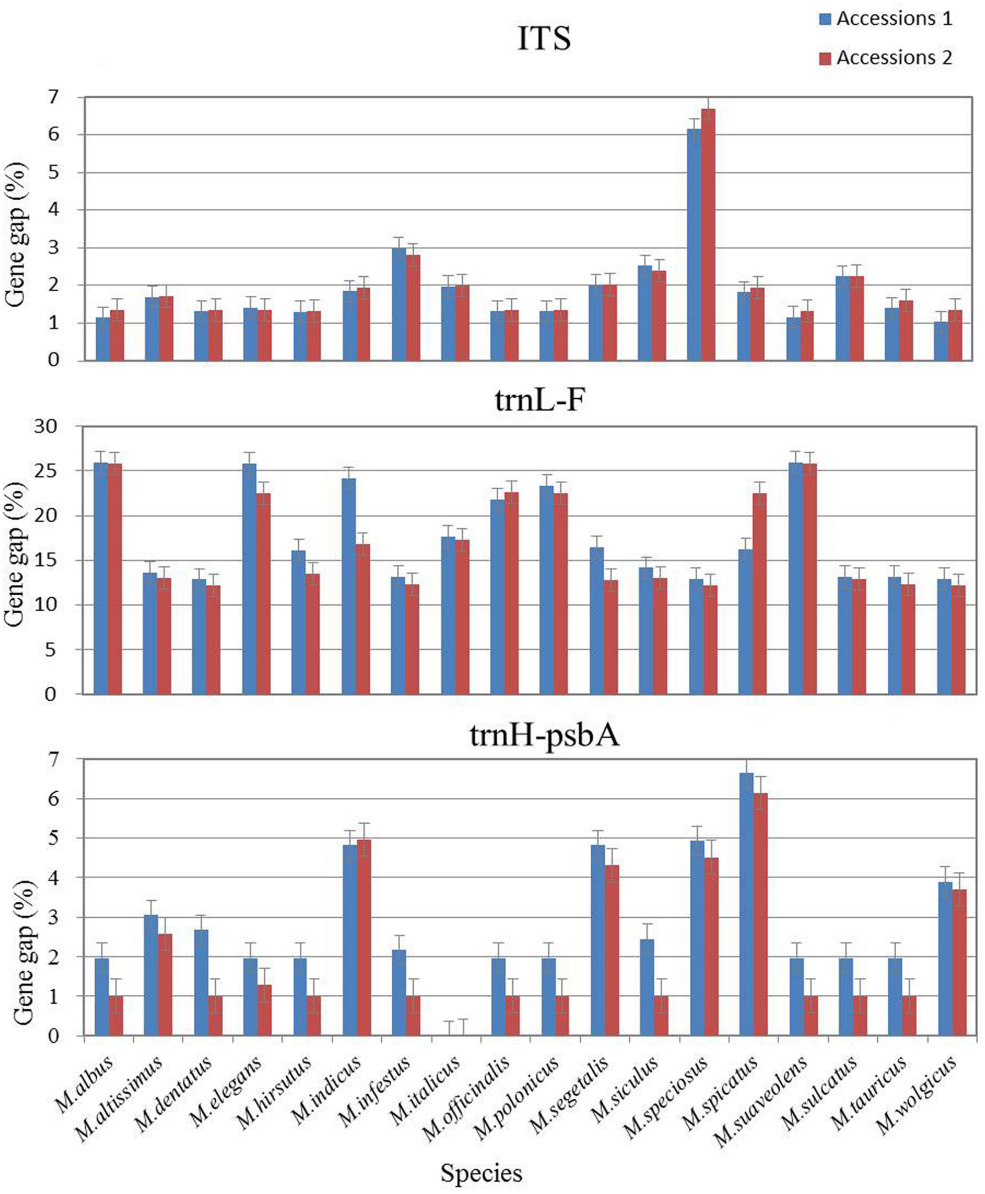
Barcode gap valued for single loci based on the analysis of 36 sequences. A total of 36 sequences from 36 plant accessions representing 18 species were examined for every DNA barcode. The plots show marginally large sequence discrepancy for different species, with sequence differences from the same species being very slight. The error bars suggest 95% confidence intervals for the probability of correct identification (PCI) estimate.

For subsamples, the percentage of species resolution ranged from 72.22% for ITS to 33.33% for *rbc*L, with 66.67% (*trn*L*)*, 52.94% (*trnH-psbA*) and 50.00% (*matK*) also observed (Fig 2). Regarding species identification, we divided the data into two subsets according to species affinity, with the following results: 13 different species were identified and gaps were similar for ITS, including *M*. *altissimus* (1.69% and 1.71%), *M*. *dentatus* (1.32% and 1.35%), *M*. *indicus* (1.85% and 1.94%) and *M*. *infestus* (2.99% and 2.83%) (Fig 3). Although a similar gap value was found for the remaining five species, the values were very close to each other. In contrast, for the *trn*L-F barcode, high gap values for *M*. *altissimus* (13.58% and 12.96%), *M*. *dentatus* (12.85% and 12.14%), *M*. *indicus* (24.15% and 16.78%), *M*. *segetalis* (16.45% and 12.81%), *M*. *suaveolens* (25.91% and 25.84%), and *M*. *wolgicus* (12.89% and 12.22%) did not allow for species identification (Fig 3). Either values that were too different for the same species or that were too similar were obtained. The result for *trn*H-*psb*A resembled those for *trn*L. Because the gap values of *mat*K and *rbc*L were zero, similarity values were employed to compare the discrimination ability of each barcode (S1 Fig), with *rbc*L exhibiting the lowest species resolution. Comparison of the data set encompassing 36 specimens using the *rbc*L barcode indicated high similarity among the species. However, the percent species resolution for the *mat*K barcode was slightly higher than that for the *rbc*L barcode (S1 Fig). Among the 18 species of *Melilotus* investigated, the ITS barcode proved to be the most appropriate by successfully discriminating 13 species, whereas *rbc*L was the least efficient, discriminating only 6 of the 18 species.

### Combination-barcode analysis

We performed several analyses to achieve a direct comparison of the identification ability of the five loci by analysing single and combination barcodes. We found that the combination of the five loci in a multi-gene trial was able to rapidly enhance species discrimination.

Overall, *mat*K+*rbc*L (MR) identified 61.11% of the species. Adding a non-coding region (*psb*A) to this combination increased the resolution to 66.67% (MRP), the same value observed for *mat*K+*rbc*L+*trn*L-F+*trn*H-*psb*A (MRPT), and the value for MRPI was even greater, at 83.33%. Moreover, 88.89% resolution, the best for discriminating among the species, was found with inclusion of all five gene regions (Fig 2).

Combining the pairwise distances of the five combined potential barcodes using at least two specimens for each was able to discriminate the 18 *Melilotus* species. The maximum and minimum interspecific distances were determined for all of the species (S4 Table). With the exception of *M*. *indicus* (7.34) and *M*. *infestus* (7.06), which were marginally lower than MRPT (7.54 and 7.24, respectively), MRTPI showed the largest pairwise distance. However, MR consistently outperformed MRP, even though the MR D-value was very small. For the five multigene combinations, MRPI values were intermediate and slightly less than those of MRTP yet significantly greater than those of MRP. Overall, the average pairwise similarity analyses (S4 Table) were not consistent with the pairwise distance. The five combinations were ranked from the most powerful to the least effective with regard to their ability to detect similarities among the species: MR>MRP>MRPI>MRTPI>MRTP.

In the multi-locus combinations, analysis of the frequency of pairwise comparisons was used to confirm the same trend in the maximum interspecific distance analysis (Fig 1B and S4 Table). The results revealed the highest frequency for MRTPI among the five combinations. The value for MRTP was followed by that for MRTPI. MR and MRP exhibited the lowest performance, despite the similar highest frequency value for each. The value of the largest frequency for MRPI was between those for MRP and MRTP (Fig 1B).

### Species identification analysis

**Fig 4:**
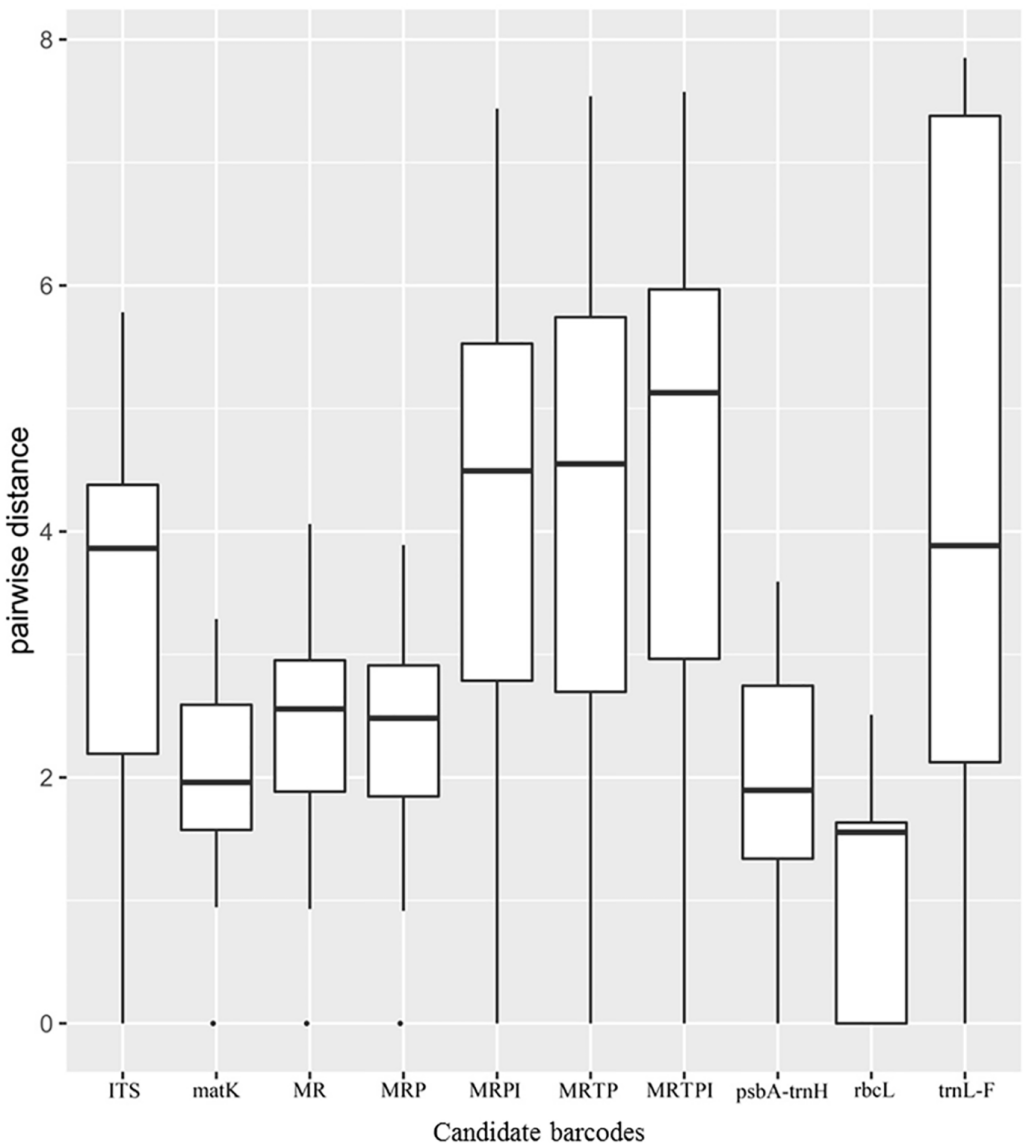
Interspecific pairwise distances analysis for each candidate barcode. All sequences of barcodes from this study were analysed with R 3.2.3. The analysis was performed to generate box plots for all possible interspecific pairwise distances for each barcode. The box plots show the combinations of barcode loci surveyed on the x axis. I, ITS; M, *mat*K; R, *rbc*L; P, *trn*H-*psb*A; T, *trn*L-F. The median is the line in the middle of the box, the upper and lower regions of the box are the 25th and 75th percentiles, respectively, and the whiskers are 1.5 times the interquartile range above and below the box limits. The dots represent outliers.

Analysis based on the pairwise distance within single and multigene barcodes was performed to generate box plots for all possible interspecific pairwise distances within each barcode. As shown in Fig 4, the biggest median value (slightly > 5) was for MRTPI. MRTP and MRPI were more effective than ITS and *trn*L-F, though the median value for all of these four barcodes was nearly four. MR and MRP exhibited a similar median value of approximately of 2.5. Moreover, *mat*K and *trn*H-*psb*A performed similarly, with smaller values (close to 2). *Rbc*L proved to be consistently the least effective marker. The variation among the pairwise differences within each barcode followed different patterns.

**Fig 5:**
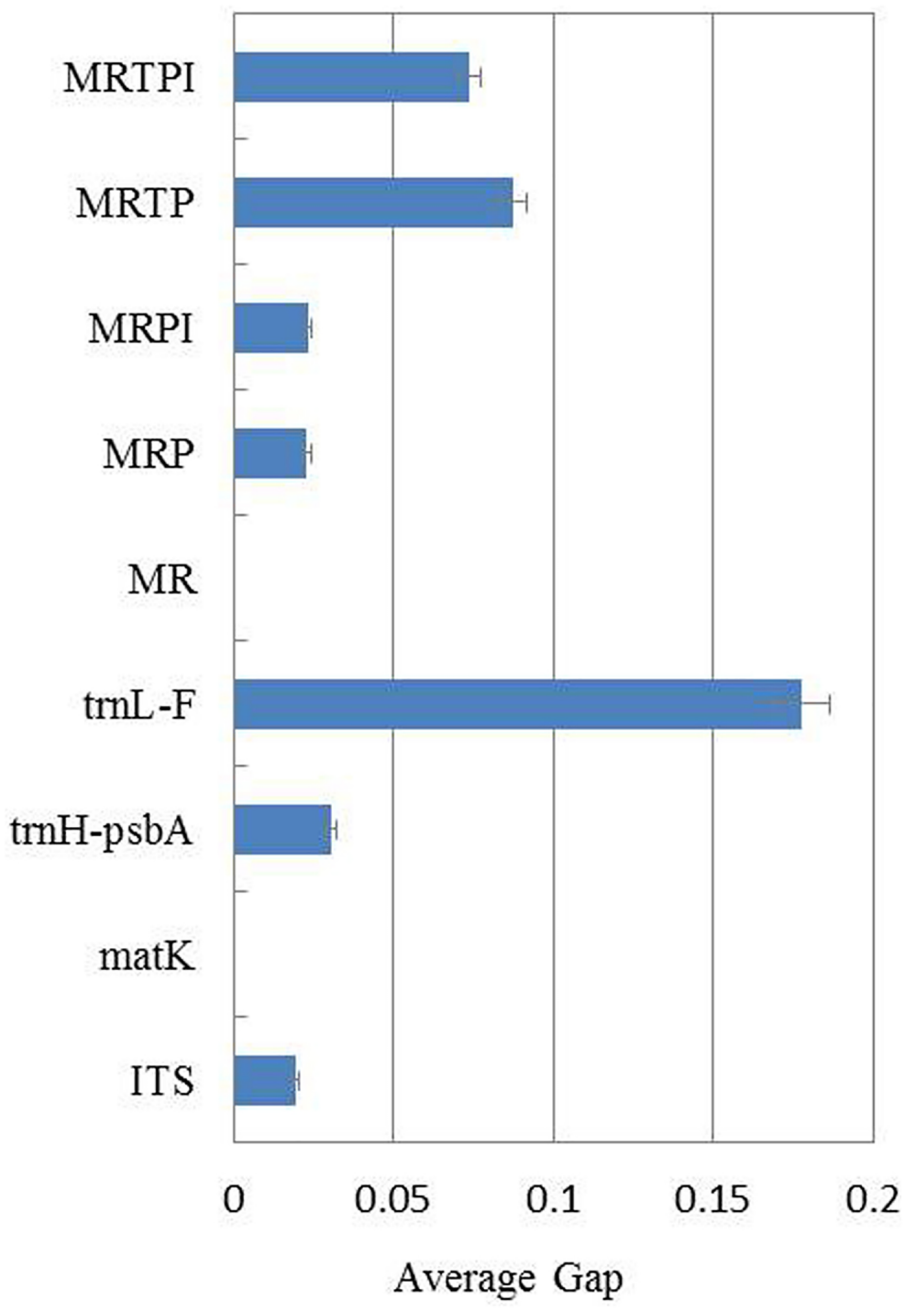
The average gap value for ten barcode systems. The plots show the combinations of barcode loci surveyed on the y axis. I, ITS; M, *mat*K; R, *rbc*L; P, *trn*H-*psb*A; T, *trn*L-F. The x axis shows the barcode gap value for *Melilotus* genus. The error bars suggest 95% confidence intervals for the barcode gap estimate.

Further analysis of the barcode gap suggested the order of *trn*L-F>MRTP>MRTPI>*trn*H-*psb*A>MRPI>MRP>ITS>MR>*mat*K for *Melilotus* (Fig 5). Ten candidate barcodes were analysed for their ability to discriminate 18 *Melilotus* species, with similar trends observed (S2 Fig). Considering all species, the largest gap value (with high marginal error) was found for *trn*L-F. The most powerful potential barcode was MRTPI, followed closely by MRTP. The same trend was observed for all species, except for *M*. *italicus*, which showed a higher gap value for MRP (0.17) than that for MRPI (0.13). Moreover, the *trn*H-*psb*A gap value for *M*. *italicus* was zero. The *rbc*L barcode exhibited the lowest identification power among all species, with a value that was consistently zero. *mat*K and MR performed poorly, as each was short of a marked barcode gap. Analysis of the barcode gap along with the score indicated a tendency toward an inverse relationship.

## Discussion

Sweet clover, valued for medicinal properties and used as animal feed, consists of 19 species mainly distributed in North and Eurasia [1]. Some species of *Melilotus* have entomophilous flowers that can result in hybridization, and it is therefore challenging to distinguish similar morphological characters among species and closely related species. Thus, a main objective of the current study was to the measure resolution rate of five potential barcodes and their combinations at the species level.

Previously, *trn*H-*psb*A is reportedly a good DNA barcode for some plants, such as orchids [41] and *Tetrastigma* (Miq.) Planch [42]. *Rbc*L and *mat*K have been proposed as plant barcodes by the CBOL Plant Working Group [25] and have also been used to discriminate species of diverse plant taxa and to clarify taxonomic origins [43, 44] in many extensive taxonomic investigations. In our study, we found that ITS was to be the best candidate barcode among the five single loci (Fig 2). Similar results have been reported in *Chlorophyta* [45] as well as higher plants [28, 46, 47]. However, the ITS region of *Ficus carica* was the l*ocus* with the lowest resolution (25.57%) but the highest variation rate (0.0188) [48], contrary to the findings of Roy who reached the best species resolution of the *Ficus* genus with this barcode [49]. ITS as a barcode exhibits high levels of inter-specific and inter-individual sequence variation because of its multi-copy nature [50]. It has been reported that ITS identification rates are the lowest and that the discrimination rate of different barcodes (ITS, *rbc*L, *mat*K, *psb*A-*trn*H) ranges from 12.21% to 25.19% in *Rhododendron* [51]. Regarding performance in species identification, the PCR amplification success rate and the sequencing success rate of ITS were very high (Table 2). Few problems in alignment or editing were observed for ITS, and the resolution far surpassed that of *rbc*L, *mat*K and *trn*H-*psb*A and slightly exceeded that of *trn*L-F (Fig 2). In general, such types of markers have greater species-resolving power; regardless, there are limitations for ITS as a standard barcode for some taxa because of amplification and sequencing difficulties [28, 42]. in addition, for a number of slowly evolving groups, genetic drift could hamper ancestral polymorphism lineage sorting [10].

Sample parameters were compared and analysed, and the results suggested that each candidate barcode was able to identify a few of the 18 species, especially ITS can identify the most of 18 species (Fig 2). As there are a series of obstacles for single markers, a suitable solution is to employ more than one marker in combination [52]. Our analysis of two-, three-, four-, or five-locus barcode systems (S4 Table) showed that MR possessed the lowest resolution, which was even lower than the single markers (ITS or *trn*L-F). However, a two-marker combination has been proposed before [53, 54], and the *rbc*L+*mat*K barcode system was able to identify 93.1% of taxa sampled from local flora [55]. A plant barcode using *rbc*L+*trn*H*-psb*A was also applied to build a library containing over 700 species of the world’s medicinal plants [56]. Although the worst identification rate of *Melilotus* species occurred with the combination of *rbc*L and *mat*K, the resolution of the assembled barcode system surpassed that of the individual barcodes. The other combinations exhibited the same patterns (Fig 2). It is reported that the combination of ITS + t*rn*H-*psb*A could enhance discrimination at 90% greater power than that of the single barcode (ITS, 70%) [57]. In *Rhododendron*, the combination of ITS+*psbA-trnH*+*matK* or ITS+*psbA-trnH*+*matK*+*rbcL* showed the highest identification rate (41.98%), far greater than a single barcode (the highest value was 25.19%) [51]. We compared the entire barcoding system performance and analysed all parameters and found that simultaneous use of the five loci was ideal for discriminating between different *Melilotus* species. A most remarkable result indicated greater resolution than with single markers, regardless of the ones combined.

In general, there are problems that limit the resolving power of barcodes, especially for chloroplast markers. Even when using all of the five barcodes together, there are small part species were identified unsuccessfully (Fig 2). Such problems can be attributed to complexity arising from reproductive and evolutionary behaviour, such as hybridization, polyploidization and mixture of sexual and asexual reproduction [47, 58]. Furthermore, in evolutionary history, introgression, reticulate evolution and incomplete lineage sorting may blur species boundaries, leading to impediments in clear barcoding [38, 59]. However, due to hybridization, it is difficult to mutate nucleotide sequences with higher conservation and synonymous substitution rates [60]. The nuclear ITS region is regarded as a core marker for identifying poisonous mushrooms [59], and our analysis confirmed that ITS is indeed a powerful potential barcode.

In conclusion, as for the whole barcode system to identify *Melilotus*, ITS was the best single candidate barcode and the assembly of five loci was the best combination candidate barcode. In addition, 18 standard barcode sequences were established for each type of barcode system in the current study, and these barcodes can be used to identify unknown *Melilotus* plants. Because hybridization and mutation always occur, discovery of novel biodiversity and efficient barcodes requires well-coordinated initiatives.

## Supporting information

S1 Fig Similarity value for single locus based on the analysis of 36 sequences. 36 sequences from 36 plant accessions representing 18 species for every DNA barcode. The plots show that sequences discrepancy of different species is marginally large and difference of sequences from the same species somehow is very slight. The error bars suggest 95% confidence intervals for the PCI estimate.
(TIF)

S2 Fig Gap value for identification of ten barcode systems in 18 *Melilotus* species. The plots show the combinations of barcode loci surveyed on the y axis. I, ITS; M, *mat*K; R, *rbc*L; P, *trn*H-*psb*A; T, *trn*L-F. The x axis shows the barcode gap value for 18 species of *Melilotus*. The error bars suggest 95% confidence intervals for the barcode gap estimate.
(TIF)

S1 Table Information for 98 accessions of 18 *Melilotus* species. (XLSX)

S2 Table Basic information and intraspecific gap and distance for 101 accessions of 18 *Melilotus* species. ITS, internal transcribed spacer.
(XLSX)

S3 Table Intra-accession gap and distance for 100 individuals of 5 *Melilotus* species. ITS, internal transcribed spacer.
(XLSX)

S4 Table Results of pairwise distance analysis based on MEGA 6.0-Compute Pairwise Distance and similarity analysis based on Emboss Needle. MR, *mat*K+*rbc*L; MRP, *mat*K+*rbc*L+*trn*H-*psb*A; MRPI, *mat*K+*rbc*L+*trn*H-*psb*A+ITS; MRTP, *mat*K+*rbc*L+*trn*L+*trn*H-*psb*A; MRTPI, *mat*K+*rbc*L+*trn*L+*trn*H-*psb*A+ITS.
(XLSX)

S1 Dataset Standardized barcode sequences of 18 *Melilotus* species for *mat*K, *rbc*L, *trn*L, *trn*H-*psb*A and ITS. (ZIP)

## Abbreviations

*CO1*: cytochrome c oxidase subunit 1
ITS: internal transcribed spacer
MR: *mat*K+*rbc*L
MRP: *mat*K+*rbc*L+*trn*H-*psb*A
MRPI: *mat*K+*rbc*L+*trn*H-*psb*A+ITS
MRTP: *mat*K+*rbc*L+*trn*L-F+*trn*H-*psb*A
MRTPI: *mat*K+*rbc*L+*trn*L-F+*trn*H-*psb*A+ITS
NJ: Neighbour Joining
NPGS: National Plant Germplasm System
PCR: polymerase chain reactions
SSR: Simple sequence repeat
