# Expression of Concern: Potential DNA barcodes for *Melilotus* species based on five single loci and their combinations

**DOI:** 10.1371/journal.pone.0230324

**Published:** 2020-04-20

**Authors:** The PLOS ONE Editors

The *PLOS* Data Availability Policy requires that, with rare prior exception, all data underlying the findings described in an article are fully available without restriction.

The Data Availability Statement for this article [1] states that all relevant data are within the paper and its Supporting Information files. The full raw sequencing data generated during this study were not deposited in an appropriate public repository at the time of publication. In an update to the Data Availability Statement, the available sequencing data used in the analyses reported in the article are deposited at Genbank. The authors have provided the Genbank accession numbers in a revised S1 Table, included in the Supporting Information.

The authors have provided the following additional information regarding the dataset:

The sequence data for a number of germplasm accessions could not be deposited because the data are missing or the information is no longer available to match the sequence data to the germplasm accession.

The dataset for this study [1] includes some of the accessions from the National Plant Germplasm System (NPGS, USA) that were analysed in a previous study [2]; the raw sequence data for these accessions were reused (specifically, raw sequences of ITS, rbcL and matK of *M*.*suaveolens* (PI593408, Ames23793), *M*. *tauricus* (Ames18446, PI67510), *M*. *dentatus* (PI108656, PI90753), *M*. *wolgicus* (PI317665, PI502547), *M*. *spicatus* (Ames18402, Ames25647), *M*. *infestus* (PI306326, PI306327), *M*. *siculus* (PI318508, PI33366), and *M*. *sulcatus* (PI198090, PI227595)). To construct the standard barcode sequence, the head and tail of each raw sequence was cut, and the trimmed sequences were used in further analyses. As the reused sequences are therefore not identical to the raw sequences generated in [2], all standard barcode sequences were submitted separately to Genbank. Thus, although the raw sequence data were reused, the Genbank sequence accessions do not overlap with those reported in [2].

For each of the 18 species, two barcode sequences were submitted to Genbank. In total, 670 sequences associated with this article [1] are deposited in Genbank. A further 204 sequences were used in the study to expand the sample sizes and increase accuracy for the analyses in Fig 4. These accession numbers are listed in the revised S1 Table and are deposited in Genbank in association with a different article [3], which has since been retracted [4].

In addition, there is an error in S1 Table of the original article. The germplasm accession PI43597 was omitted from the table. Please see the revised S1 Table here.

The *PLOS ONE* Editors issue this Expression of Concern to alert readers that the underlying sequence data for the article are not available in full, which affects the reproducibility of the reported analyses.

## Supporting information

S1 Table Information for 98 aCCESsions of 18 *Melilotus* species. (XLSX)
