# Supplementary figures and images for: Potential DNA barcodes for Melilotus species based on five single loci and their combinations
Source: PLoS One. 2017 Sep 14;12(9):e0182693. doi: 10.1371/journal.pone.0182693 (PMC5598934; doi:10.1371/journal.pone.0182693)

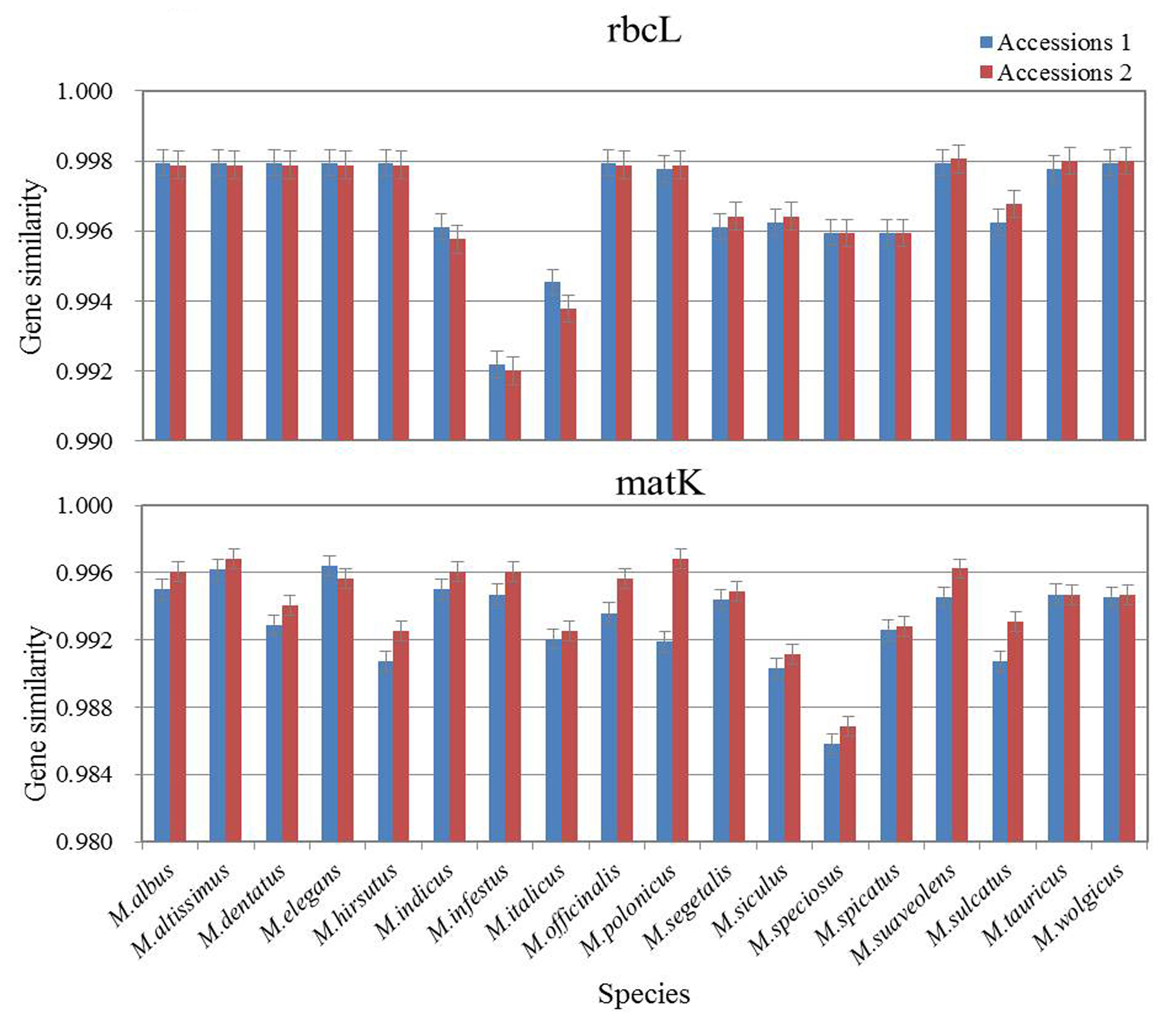

Supplement: S1 Fig — 36 sequences from 36 plant accessions representing 18 species for every DNA barcode. The plots show that sequences discrepancy of different species is marginally large and difference of sequences from the same species somehow is very slight. The error bars suggest 95% confidence intervals for the PCI estimate. (TIF) [file pone.0182693.s001.tif]

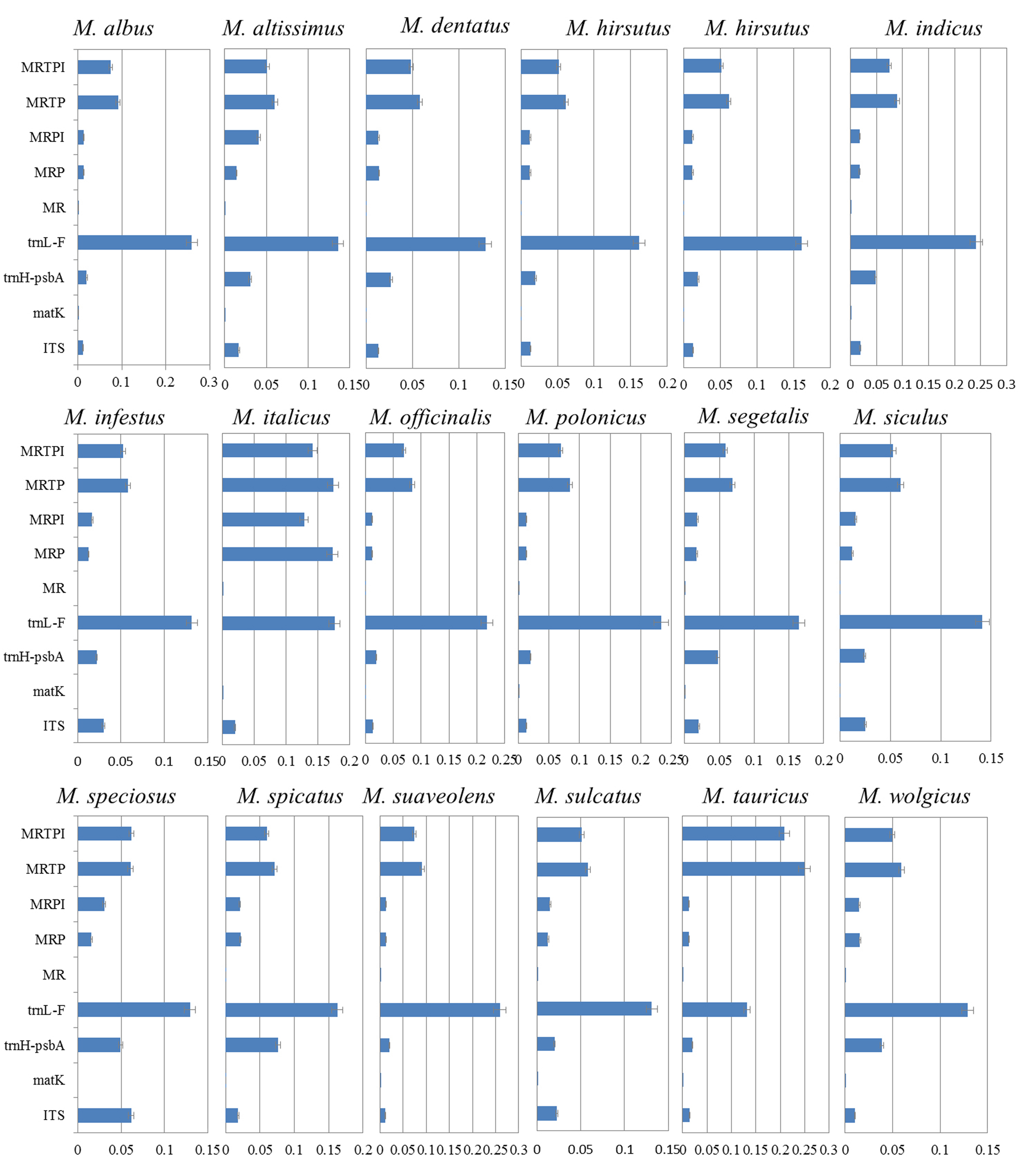

Supplement: S2 Fig — The plots show the combinations of barcode loci surveyed on the y axis. I, ITS; M, matK; R, rbcL; P, trnH-psbA; T, trnL-F. The x axis shows the barcode gap value for 18 species of Melilotus. The error bars suggest 95% confidence intervals for the barcode gap estimate. (TIF) [file pone.0182693.s002.tif]
